# Biochanin A Reduces Inflammatory Injury and Neuronal Apoptosis following Subarachnoid Hemorrhage via Suppression of the TLRs/TIRAP/MyD88/NF-*κ*B Pathway

**DOI:** 10.1155/2018/1960106

**Published:** 2018-06-03

**Authors:** Ling-yun Wu, Zhen-nan Ye, Zong Zhuang, Yongyue Gao, Chao Tang, Chen-hui Zhou, Chun-xi Wang, Xiang-sheng Zhang, Guang-bin Xie, Jing-peng Liu, Meng-liang Zhou, Chun-hua Hang, Ji-xin Shi

**Affiliations:** ^1^Department of Neurosurgery, Jinling Hospital, School of Medicine, Nanjing University, 305 East ZhongShan Road, Nanjing, Jiangsu Province 210002, China; ^2^Department of Neurosurgery, the Second Affiliated Hospital of Guangzhou Medical University, Guangzhou, Guangdong Province 510260, China; ^3^Department of Neurosurgery, Jinling Hospital, School of Medicine, Southern Medical University (Guangzhou), Nanjing, Jiangsu Province 210002, China

## Abstract

Inflammatory injury and neuronal apoptosis participate in the period of early brain injury (EBI) after subarachnoid hemorrhage (SAH). Suppression of inflammation has recently been shown to reduce neuronal death and neurobehavioral dysfunction post SAH. Biochanin A (BCA), a natural bioactive isoflavonoid, has been confirmed to emerge the anti-inflammatory pharmacological function. This original study was aimed at evaluating and identifying the neuroprotective role of BCA and the underlying molecular mechanism in an experimental Sprague-Dawley rat SAH model. Neurobehavioral function was evaluated via the modified water maze test and modified Garcia neurologic score system. Thus, we confirmed that BCA markedly decreased the activated level of TLRs/TIRAP/MyD88/NF-*κ*B pathway and the production of cytokines. BCA also significantly ameliorated neuronal apoptosis which correlated with the improvement of neurobehavioral dysfunction post SAH. These results indicated that BCA may provide neuroprotection against EBI through the inhibition of inflammatory injury and neuronal apoptosis partially via the TLRs/TIRAP/MyD88/NF-*κ*B signal pathway.

## 1. Introduction

Subarachnoid hemorrhage (SAH) is known as an acute catastrophic neurological disease with a high rate of perioperative mortality and permanent morbidity worldwide. Currently, at least a quarter of SAH patients pass away instantly and more than half of SAH survivors endure the pain of the severe long-term neurological deficits [1]. Recent basic and clinical studies have focused on the pathologic mechanism that occurs in the period from 0 h to 72 h post SAH, often known as early brain injury (EBI), the most crucial and etiological factor of poor clinical prognosis among SAH cases [2]. In recent years, excessive inflammatory activity and neuronal apoptosis post SAH have been found in clinical cases and animal experiments [3]. In addition, the degree of secondary inflammatory injury and severe neuronal apoptosis correlates with the progress of SAH, and many previous studies implicated that the uncontrolled inflammatory response and neuronal apoptosis may aggravate EBI post SAH [4].

Biochanin A (BCA), an organic isoflavone derived from natural plant sources, has been classified as a special phytoestrogen [5–7]. BCA has exhibited various beneficial bioactivities, such as antidiabetic, anticancer, antiallergic, and anti-inflammatory effects [8]. Recent studies demonstrated that BCA can provide affirmative neuroprotection against the cerebral ischemia/reperfusion-induced injury [9]. In addition, BCA attenuates lipopolysaccharide (a TLR-mediated pathway agonist, LPS)-induced inflammatory response and the transcription of NF-*κ*B [10–14]. Moreover, we have previously found that TLR/NF-*κ*B pathway-mediated inflammatory response and neuronal apoptosis participate in the EBI period post SAH [15, 16]. However, the neuroprotective role of BCA in EBI has not been studied so far. Above all, all these extend naturally to our hypothesis that BCA administration may provide neuroprotection via inhibiting the TLR/TIRAP/MyD88/NF-*κ*B-related inflammation and reducing neuronal apoptosis after SAH in rats.

## 2. Methods

### 2.1. Experimental Sprague-Dawley Rats and BCA Administration

Experimental Sprague-Dawley rats (weighted 300 ± 20 g) were obtained from the experimental management animal center of Nanjing University, Jiangsu, China. All Sprague-Dawley rats were maintained under standard animal laboratory circumstances for one week before the experimental protocols. BCA powder reagent was obtained from Sigma Chemicals, USA, and dissolved in dimethyl sulfoxide (DMSO, as a vehicle, final concentration: 0.5% DMSO in 0.9% normal saline). BCA was given intracerebroventricular injection to rats at 1 h after the induction of SAH at a dosage of 10 *μ*g/kg, 20 *μ*g/kg, and 40 *μ*g/kg.

All the experimental protocols including animal drug administration and animal surgical procedures were approved by the Institutional Animal Care and Use Committee of Nanjing University (Jinling Hospital) and were consistent with the guidelines for the Care and Use of Laboratory Animals by National Institutes of Health (China).

### 2.2. Rat SAH Model

We used prechiasmatic interspace injection (300 *μ*l per rat) to build this rat SAH model according to our previous study [17]. In addition, 300 *μ*l normal saline solution was given to the sham group. Rats that died during the experimental period were abandoned, and we wound repeated the surgery to make sure every group could get the planned sample size number.

### 2.3. Experimental Design and Tissue Preparation Time Point

**Figure 1:**
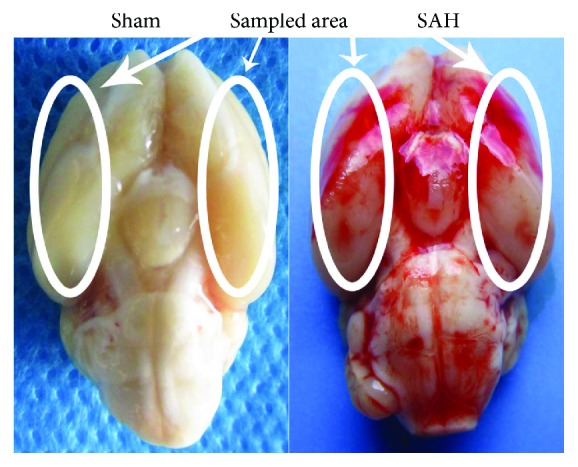
Sample collection location and rat SAH model.

A total 172 of rats were divided into 7 groups randomly as follows: sham group (*n* = 32), sham + BCA 20 *μ*g/kg group (*n* = 20), SAH group (*n* = 32), SAH + vehicle group (*n* = 32), SAH + BCA 10 *μ*g/kg group (*n* = 12), SAH + BCA 20 *μ*g/kg group (*n* = 32), and SAH + BCA 40 *μ*g/kg group (*n* = 12). According to the results from previous studies on SAH-induced inflammation and neuronal apoptosis, we choose the 24 h after SAH for sample collection [18, 19]. Respectively, 132 rats in the abovementioned groups were euthanized for sample tissue preparation, sample obtained location, and tissue preparation method according to our previous study [16] (Figure 1). The left rats in part of groups were prepared to do the modified water maze test.

### 2.4. Brain Water Content

Left hemisphere brain tissue, right hemisphere brain tissue, cerebellum tissue, and brain stem tissue from rat brain tissue were dried in 100°C for three days to get the brain dry weight data after weighing for wet data. [16].

### 2.5. Western Blot Reagent

Primary antibodies were as follows: myeloid differentiation factor 88 (MyD88, Santa Cruz, USA, 1 : 200–1 : 400), TIR domain-containing adaptor protein (TIRAP, Abcam, USA, 1 : 1000), TLR4 (Santa Cruz, USA, 1 : 200–1 : 400), TLR2 (Santa Cruz, USA, 1 : 200–1 : 400), Iba1 (anti-IBA, ab15690, Abcam, USA, 1 : 500, active microglial marker), NF-*κ*B P65 (Santa Cruz, USA, 1 : 200–1 : 400), Bax (Santa Cruz, USA, 1 : 200–1 : 400), Bcl-2 (Santa Cruz, USA, 1 : 200–1 : 400), caspase-3 (Bioss, Beijing, China, 1 : 100–1 : 500). Histone 3 (anti-H3, ab1791, Abcam, USA, 1 : 1000, nuclear loading control) and *β*-actin (1 : 5000, Bioworld Technology, USA) were used as a loading control in nuclear protein and total protein. After conjunction with the HRP-secondary antibody (1 : 1000, Bioworld Technology, USA), the target protein bands were visualized and recorded by Tanon-5200 Chemiluminescent Imaging System [20].

### 2.6. Enzyme-Linked Immunosorbent Assay (ELISA) and Electrophoretic Mobility Shift Assay (EMSA)

The ELISA assay kit system for rats (Affymetrix eBioscience, Santa Clara, USA) was used to evaluate the concentration of TLR downstream productive cytokines in a total protein sample [16]. The EMSA Assay kit System (Affymetrix eBioscience, Santa Clara, USA) was used to evaluate the degree of NF-*κ*B/DNA-combining activity [16].

### 2.7. TUNEL Immunofluorescence Staining

Immunofluorescence (IF) staining was operated to detect the TUNEL-positive neurons (anti-NeuN, Alexa Fluor® 488 conjugated, Millipore, USA, 1 : 100) and the immunoreactivity of Iba1 (anti-IBA, ab15690, Abcam, USA, 1 : 100, active microglial marker) [21]. Apoptosis was determined by TUNEL detection kit (TMR red immunofluorescence staining kit from Sigma-Aldrich, MO, USA). Positive-cell counting and statistical analysis were restricted to our previous study [22]. The percentage of TUNEL-positive cells was calculated as follows: (number of TUNEL − positive neurons/total number of neurons) × 100%.

### 2.8. Neurologic Scoring and Spatial Learning and Cognitive Memory Testing

We used the modified Garcia score system to assess the rats' neurological changes according to our previous study [23]. Spatial learning and cognitive memory ability were assessed via a modified water maze test [16]. Data from the modified water maze test were as follows: escape latencies (s, time data and average data) and swimming distance (cm, distance data and average data) in every test trail for the rats to get on the hidden platform in the water maze [24].

### 2.9. Statistical Analysis

All the experimental data in this study was presented as mean value ± SD score. SPSS 19.0 was used for statistical analysis and calculation. All the data was subjected to one-way ANOVA comparison test. Values of ^∗^*p* and ^#^*p* < 0.05 were considered data statistically significant.

## 3. Results

### 3.1. General Mortality and Sample Collection Location

The total rate of mortality in the sham group was 0% (0/32 rats), that in the sham + BCA group was 0% (0/20), and that in the SAH-processed group was 21.57% (33/153 rats). As shown in Figure 1, we collected samples at the locations shown in Figure 1 in each group at 24 h after the establishment of SAH for the further bimolecular analyses performed.

### 3.2. BCA Improves the Rats' Neurological Score and Reduces Cerebral Hemisphere and Cerebellum Edema at 24 h Post SAH

**Figure 2:**
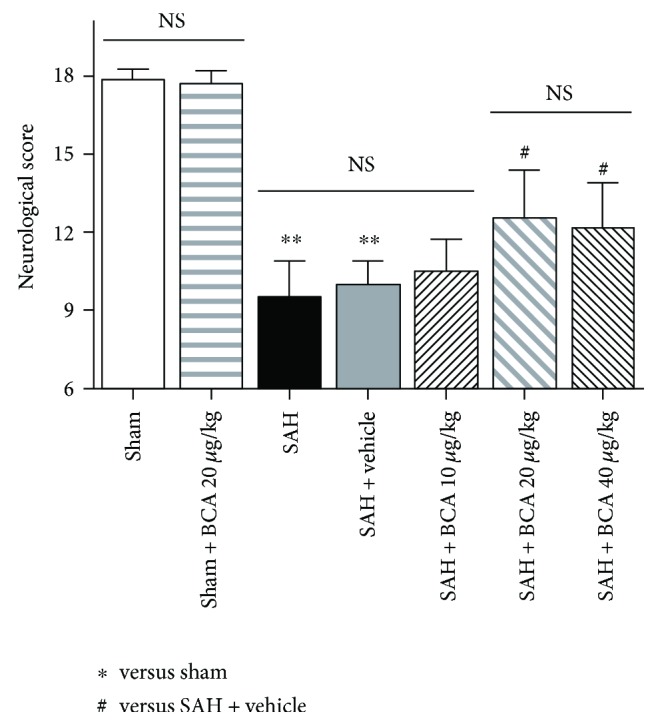
The effects of BCA on neurological function at 24-hour post-SAH rats and sham rats. (*n* = 6, each group; ^∗∗^*P* < 0.01 versus sham; ^#^*P* < 0.05 versus SAH + vehicle; ^NS^*P* > 0.05).

**Figure 3:**
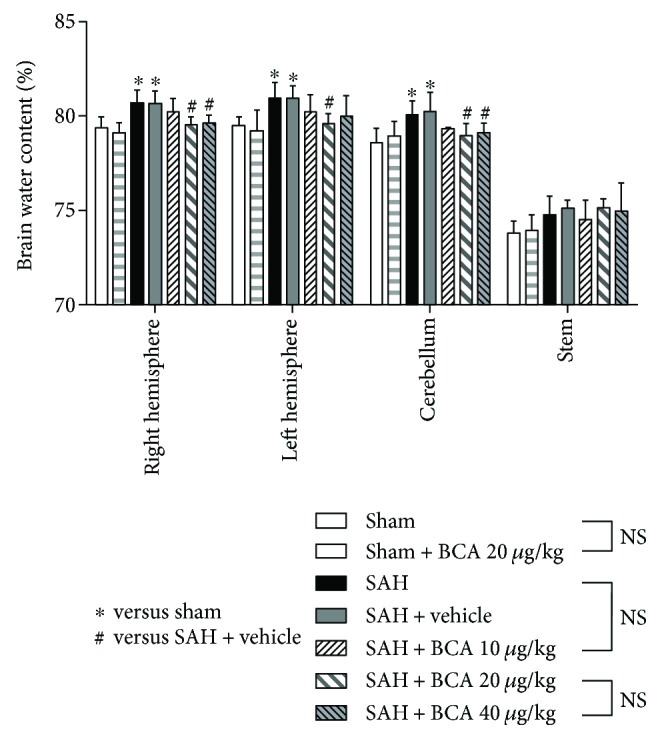
The effects of BCA on the brain water content levels in the left hemisphere, right hemisphere, cerebellum, and brain stem at 24 h post SAH (*n* = 6, each group; ^∗^*P* < 0.05 versus sham; ^#^*P* < 0.05 versus SAH + vehicle; ^NS^*P* > 0.05).

In this part, modified Garcia score and rats' right and left hemisphere, cerebellum, and stem water content were recorded and evaluated. As shown in Figures 2 and 3, treatment with BCA at 20 *μ*g/kg and 40 *μ*g/kg both improved the modified Garcia score and reduced cerebral hemisphere and cerebellum edema at 24 hours post SAH. However, the higher dose (40 *μ*g/kg) did not bring a better therapeutic effect than the dose at 20 *μ*g/kg did. Meanwhile, BCA at 20 *μ*g/kg did not produce the side effect of neurological toxicity on sham rats. Therefore, 20 *μ*g/kg was selected as the best treatment dose for the further mechanism study and spatial learning/cognitive memory test.

### 3.3. BCA Reduced the Cell Number of Active Microglia at 24 Hours Post SAH

**Figure 4:**
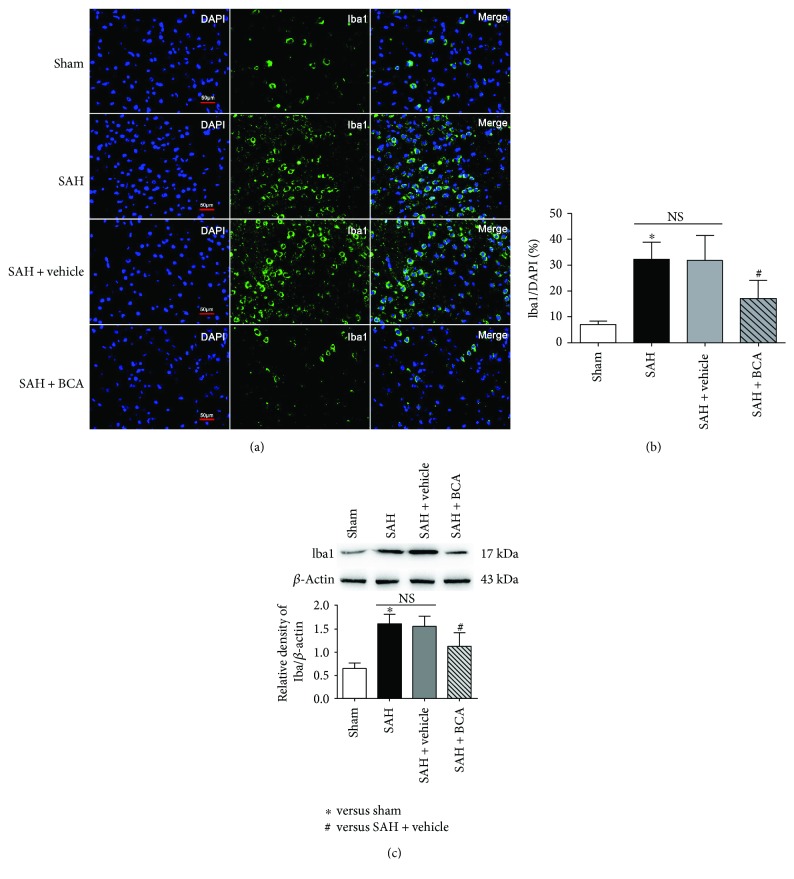
The effects of BCA on microglial activation at 24 h post SAH. (a, b) Immunofluorescence staining: Iba1 = green, DAPI = blue, scale bars = 50 *μ*m. (c) Western blot analysis.(*n* = 6, each group; ^∗^*P* < 0.05, versus sham; ^#^*P* < 0.05 versus SAH + vehicle; ^NS^*P* > 0.05).

We use the ionized calcium-binding adaptor molecule 1 (Iba-1) as the marker of activated microglia; the percent of Iba-1-positive microglia increased after the SAH operative process. BCA administration at 20 *μ*g/kg reduced the percentage of active microglia which demonstrated its potential anti-inflammatory effects in this SAH model study. The percentage was calculated as follows: (number of Iba1 − positive microglia/total number of DAPI) × 100% (Figure 4).

### 3.4. BCA Suppressed the Levels of TLR2/TLR4/TIRAP/MyD88 Pathway Protein and NF-*κ*B/DNA-Combining Activity at 24 Hours Post SAH

**Figure 5:**
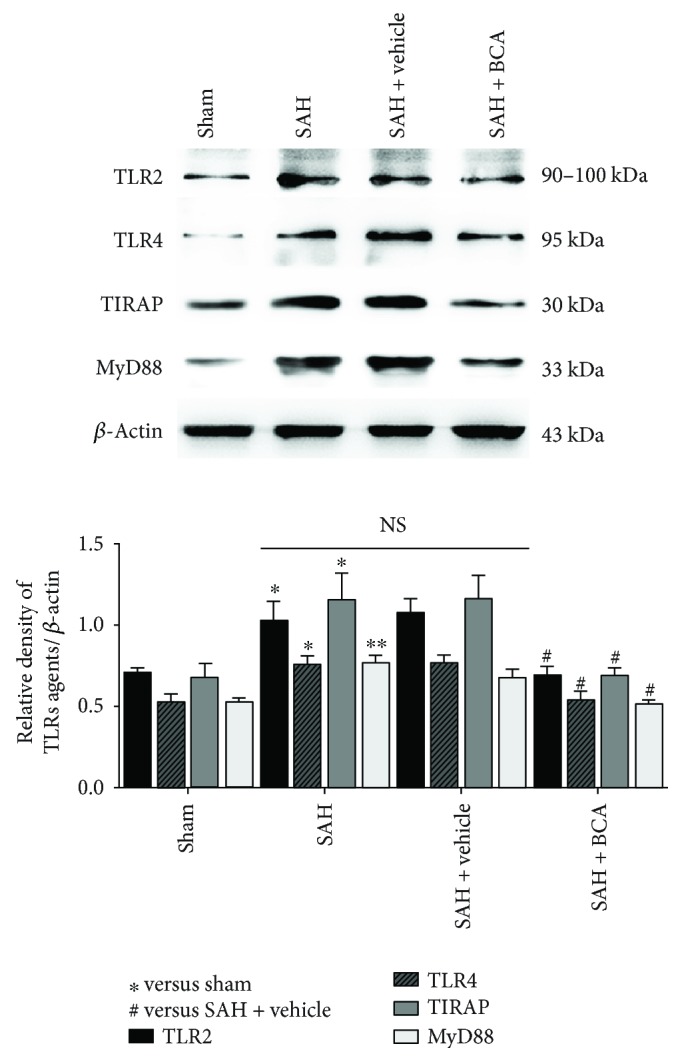
The effects of BCA on TLR2/TLR4/TIRAP/Myd88 signal protein expression at 24 hours post SAH (*n* = 6, each group; ^∗^*P* < 0.05 and ^∗∗^*P* < 0.01 versus sham; ^#^*P* < 0.05 versus SAH + vehicle; ^NS^*P* > 0.05).

**Figure 6:**
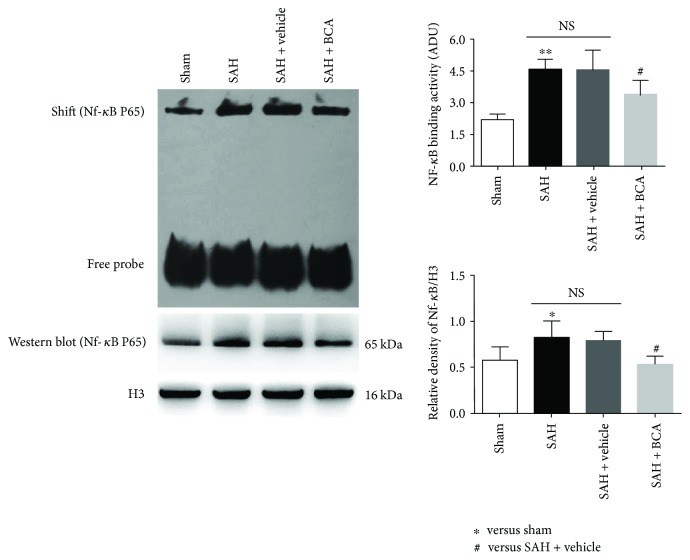
BCA administration suppressed NF-*κ*B/DNA-combining activation and the nuclear level of NF-*κ*B p65 at 24 hours post SAH (*n* = 6, each group; ^∗^*P* < 0.05 versus sham; ^∗∗^*P* < 0.01 versus sham; ^#^*P* < 0.05 versus SAH + vehicle; ^NS^*P* > 0.05).

The pathway-related proteins were suppressed via the administration of BCA versus vehicle treatment rats at 24 hours post SAH (Figure 5, Western blot). Lower DNA-combining activity and lower nuclear transfer of NF-*κ*B were found in the SAH + BCA group versus vehicle treatment rats at 24 hours post SAH (Figure 6, EMSA).

### 3.5. BCA Decreases the Cytokine Release in Rats' Brain at 24 Hours Post SAH

**Figure 7:**
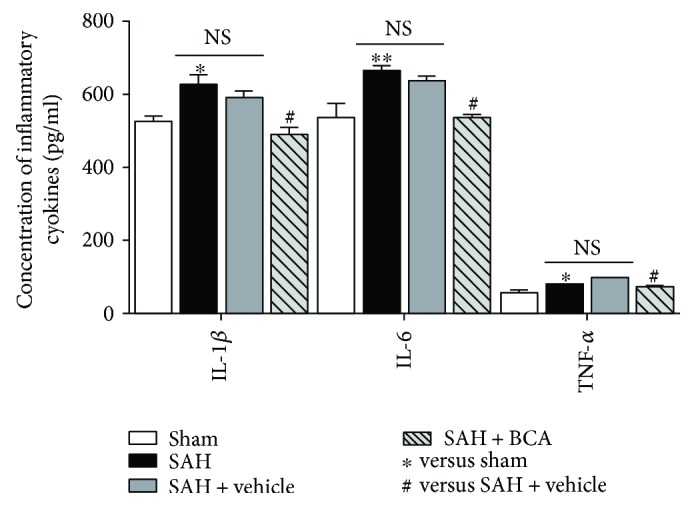
The effects of BCA on the concentrations of inflammatory cytokines at 24 hours post SAH (*n* = 6, each group; ^∗^*P* < 0.05 and ^∗∗^*P* < 0.01 versus sham; ^#^*P* < 0.05 versus SAH + vehicle; ^NS^*P* > 0.05).

According to the results of ELISA, with the administration of BCA, the concentrations of inflammation-related cytokines in rats' brain tissue were lower than in the vehicle treatment group in this study. This is obviously the certain consequence of inhibition of NF-*κ*B transcription-related cytokine release (Figure 7).

### 3.6. BCA Modulated the Apoptosis-Related Functional Proteins and Reduced the Percent of Neuronal Apoptotic Death at 24 Hours Post SAH

**Figure 8:**
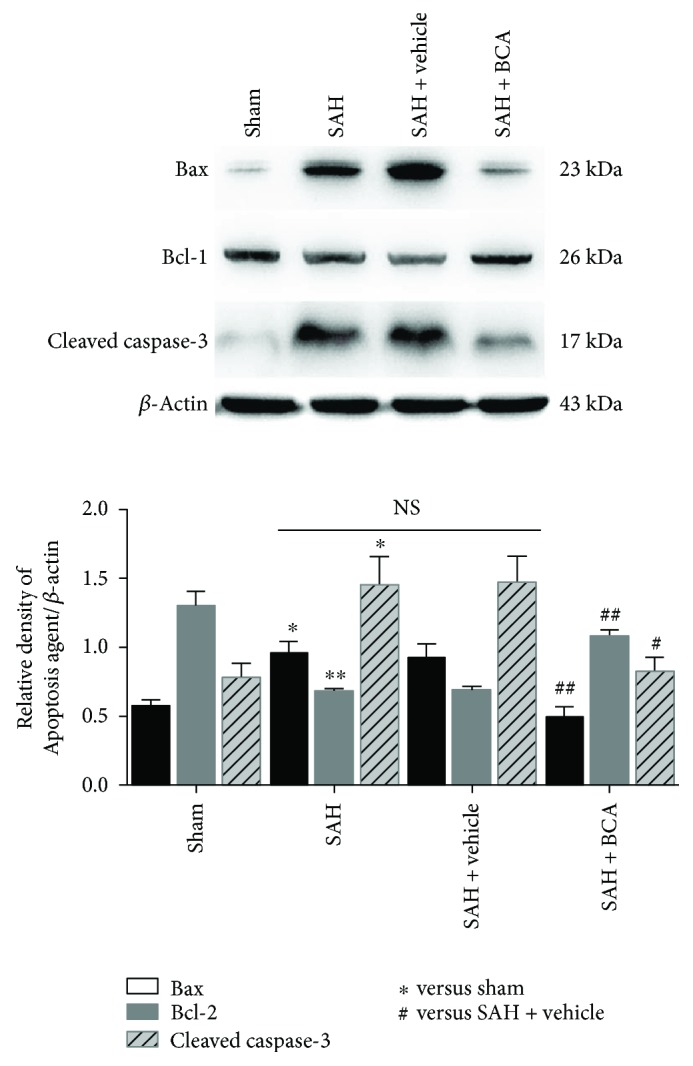
BCA administration suppressed the apoptotic process at 24 hours post SAH (*n* = 6, each group; ^∗^*P* < 0.05 and ^∗∗^*P* < 0.01 versus sham; ^#^*P* < 0.05 and ^##^*P* < 0.01 versus SAH + vehicle; ^NS^*P* > 0.05).

**Figure 9:**
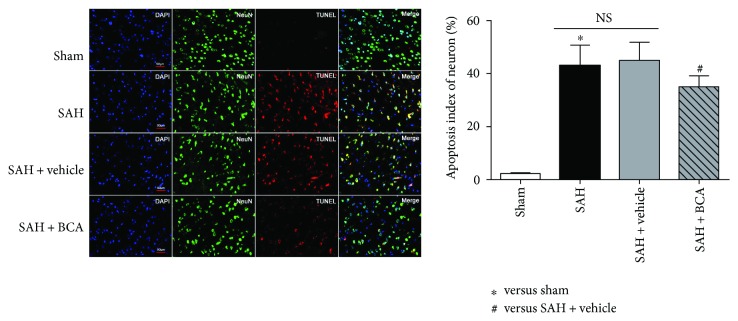
BCA attenuates the level of TUNEL-positive neurons at 24 hours post SAH. (TUNEL = red, NeuN = green, DAPI = blue, scale bars 50 *μ*m *n* = 6, each group; ^∗^*P* < 0.05 versus sham; ^#^*P* < 0.05 versus SAH + vehicle; ^NS^*P* > 0.05).

BCA treatment downregulated the levels of Bax (proapoptosis) and cleaved caspase-3 (proapoptosis) and upregulated the expression of Bcl-2 (antiapoptosis) versus the vehicle treatment group (Figure 8). As a consequence, the percentage of TUNEL (red)-positive neurons (green) in the BCA treatment group reduced to less than 40% versus more than 40% in vehicle treatment rats (Figure 9).

### 3.7. BCA Improved SAH Rats' Spatial Learning and Cognitive Memory Dysfunction

**Figure 10:**
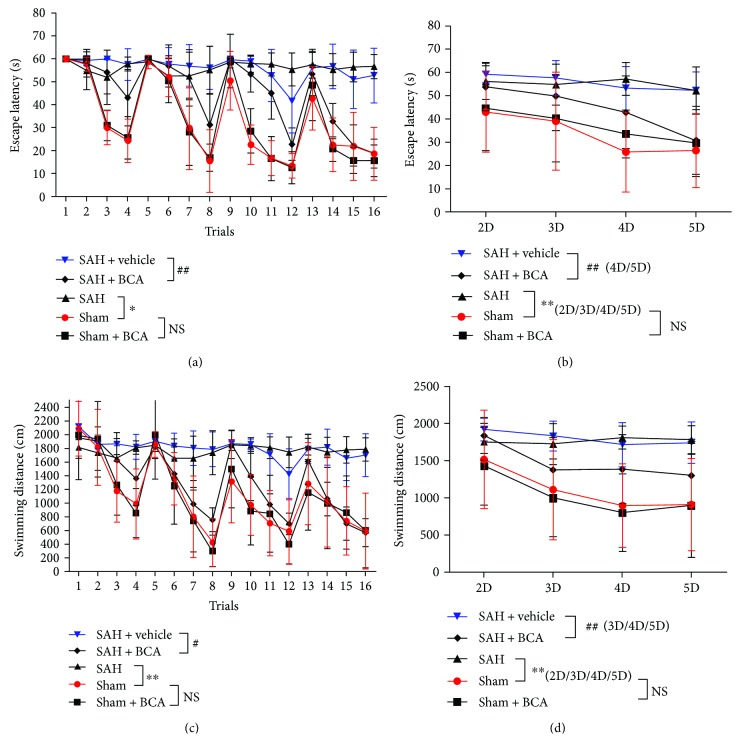
BCA reduced the escape latency (a, *n* = 8, ^∗^*P* < 0.05 and ^##^*P* < 0.01, one-way ANOVA) and traveled distance (c, *n* = 8, ^∗∗^*P* < 0.01 and ^#^*P* < 0.05) in 16 trials post SAH. In addition, the averaged data showed significantly shorter escape latency (b, *n* = 8, ^∗∗^*P* < 0.01 and ^##^*P* < 0.01, one-way ANOVA) on day 4 and day 5 and traveled distance (d, *n* = 8, ^∗∗^*P* < 0.01 and ^##^*P* < 0.01, one-way ANOVA) on day 3, day 4, and day 5 in the BCA treatment group.

In the Morris water maze test, each rat was tested for 4 trials/day from day 2 to day 5 after the induction of SAH (Figures 10(a)–10(d)). Data analysis indicated that SAH rats exhibited severe spatial learning and cognitive memory dysfunction; many experiment objects in the SAH group showed a blind search movement during the 4-day test. What is surprising is that BCA treatment facilitated SAH rats to get the hidden platform in a shorter time period and path distance versus the vehicle group (Figures 10(a) and 10(c)). The averaged data showed the same phenomenon of improvement (Figures 10(b) and 10(d)) via BCA treatment versus the vehicle treatment group. In summary, BCA improved rats' spatial learning and cognitive memory dysfunction after SAH and exhibited no side effects on sham rats.

## 4. Discussion

BCA has been considered an anti-inflammatory, antioxidative, and antiproliferative compound in many previous studies [5, 25–27]. According to our results, we found that the dosage at 20 *μ*g/kg and 40 *μ*g/kg BCA improved the neurological dysfunction and ameliorated cerebral edema post SAH. A further increase in the dosage may exceed the ability of the cerebrospinal fluid to deliver the drug, which led to no better therapeutic effect at a higher dosage (40 *μ*g/kg). Therefore, we selected 20 *μ*g/kg as the best effective therapeutic concentration in CSF for treatment. A more reasonable route of administration and time window for treatment is still worthy of further exploration. In addition, BCA at 20 *μ*g/kg did not have an effect on sham rats in brain water content, neurological function scores, and spatial learning and cognitive memory ability. These data suggested that BCA might serve as one ideal therapeutic medicine for SAH treatment without apparent side effects.

The toll-like receptor (TLR) family owns the responsibility of initiating innate immunity response including inflammatory response in the stress status [28]. In addition, MyD88 and TIRAP act as binding proteins among TLR-mediated immunity response activity cases [29–31]. TLRs, MyD88, and TIRAP form a complex network structure of the TLR-mediated inflammatory cascade. When SAH occurred and blood flowed into the subarachnoid space, the cleavage products released from cracked blood cells (especially red blood cells) could lead to the activation of a TLR/MyD88/TIRAP signal complex so-called pathogens, which could initiate inflammatory cascade response and then damage neurons and other functional cells [32, 33]. In addition, the nuclear factor NF-*κ*B, as the most important downstream transcription factor following the TLR-mediated signal complex, was closely associated with the release of cytokines [34]. Moreover, in inflammatory injury cases, an acute excess production or release of inflammatory cytokines could exert potential harmful effects on the tissue system [35]. According to our previous studies, inhibiting the TLR/NF-*κ*B cascade pathway could show therapeutic effects on EBI following SAH, which means that the TLR4/NF-*κ*B pathway could be an important therapeutic target in SAH treatment [36]. In this SAH model research, we confirmed our hypothesis that BCA inhibited the expression of TLR2/4/MyD88/TIRAP/NF-*κ*B signal axis protein and reduced the production and release of cytokines post SAH. It is noteworthy that the inhibitory effect of BCA on the TLR/NF-*κ*B pathway is consistent with its antineuronal apoptosis and anti-TLR-mediated-inflammatory effect and therapeutic effect on neurological dysfunction. Furthermore, BCA relieved the severity of brain edema, rendering that the role of BCA in blood-brain barrier functional protection is worthy of further study. Our study results revealed that BCA inhibited the TLR/MyD88/TIRAP/NF-*κ*B-signaling pathway while alleviating EBI, suggesting that suppression of the TLR/MyD88/TIRAP/NF-*κ*B-signaling pathway at least may be one of the mechanisms under its therapeutic effect.

According to published clinical research, nearly more than half of SAH survivors have suffered cognitive awareness dysfunction and neurological disorder [37]. In the present study, rats suffering experimental SAH showed apparent spatial learning and cognitive memory deficits, which were visualized and investigated in the modified Morris water maze test [37]. Moreover, one recent study found that BCA improves cognitive deficit in the ischemic stroke mice model [38]. We are pleased to verify the same therapeutic effect of BCA on rats' spatial learning and memory deficits in this model of SAH, along with the downregulation of the apoptotic process and inflammatory response. In summary, BCA may exhibit a great potential in understanding and treating cognitive or neurobehavioral deficits post SAH due to its anti-TLR/NF-*κ*B-mediated inflammatory and antineuronal apoptotic effects.

In this study, we speculated that BCA could alleviate the inflammatory responses contributing to EBI post SAH. However, the signal network of inflammation-related injury and neuronal apoptosis in EBI post SAH is very complicated and intricate. Multiple independent proteins and intricate signaling pathways may participate in the therapeutic effects of BCA. In this study, we cannot exclude the possibilities that BCA may protect against EBI via some other inflammatory signal pathway. Hence, further studies are warranted to address these issues. We hope our current research will help to further demonstrate the role and value of BCA in cerebrovascular disease, especially in SAH study.

## 5. Conclusions

In conclusion, our present data indicate that BCA provides neuroprotection against EBI possibly via suppressing TLR/NF-*κ*B pathway-mediated inflammatory agents and suppressing excessive neuronal apoptosis post SAH. Consequently, BCA further supports its protective role as a potential pharmacological reagent to reduce EBI post SAH.

## Data Availability

The data used to support the findings of this study are available from the corresponding author upon request.
